# Development of purple-grain triticale

**DOI:** 10.18699/vjgb-26-64

**Published:** 2026-07

**Authors:** N.V. Petrash, P.I. Stepochkin

**Affiliations:** Institute of Cytology and Genetics of the Siberian Branch of the Russian Academy of Sciences, Novosibirsk, Russia Siberian Research Institute of Plant Production and Breeding – Branch of the Institute of Cytology and Genetics of the Siberian Branch; Institute of Cytology and Genetics of the Siberian Branch of the Russian Academy of Sciences, Novosibirsk, Russia Siberian Research Institute of Plant Production and Breeding – Branch of the Institute of Cytology and Genetics of the Siberian Branch

**Keywords:** triticale, purple grain color, marker-assisted selection, anthocyanin, тритикале, фиолетовая окраска зерна, маркер-вспомогательная селекция, антоцианы

## Abstract

Purple-colored grains of cereal crops are characterized by high antioxidant activity. Anthocyanins, polyphenolic compounds found in the pericarp of their grains, have beneficial effects on human health. However, triticale has not yet developed forms with anthocyanin-rich purple grain color. The aim of this work was to obtain new forms of wheat-rye amphiploids with purple grain color using marker-assisted breeding and to compare their anthocyanin content and productivity indicators. Molecular DNA markers were used to determine the genotype of hybrids produced by triticale Sadko (× Triticosecale Wittmack) and a purple-colored emmer wheat line 27-3/17 (T. dicoccum (Schrank) Schuebl.). Purple-colored F3 hybrids carried two complementary dominant genes Pp3 and Pp-B1 in a homozygous state responsible for the high content of anthocyanins in the grain. In subsequent generations, the wheat-rye amphiploids had a purple grain color. The total anthocyanin content in the whole grain flour of the hybrids ranged from 36 to 529.3 μg/g. The high content was recorded in sample 2-1-6-6 (529.3 μg/g). Samples 2-1-1-4e, 2-1-5-10a and 2-1-6-4b were at the control level (emmer wheat – 382.6 3 μg/g). The F5–6 hybrid plants had a typical hexaploid triticale phenotype. The spike length and the number of spikelets exceeded those of emmer wheat. The number of spike grains in the hybrids was less than that in the Sadco triticale averaging at 28.0 and 34.4 in 2024 and 2025, respectively. 1,000 grain weight of purple-grained triticale families in 2025 was comparable to the Sadko maternal form and averaged 47.6 g. The yield per unit area of hybrid families (470 g/ m2) in 2025 was higher than that of emmer wheat (306 g/m2), but lower than that of Sadko (584 g/m2). Thus, the breeding material of purple-grained triticale forms was obtained, which in a number of ways is similar to the triticale Sadko maternal form, but differs from the paternal form of the purple grain donor emmer wheat.

## Abstract (ru)

Зерна злаковых культур с фиолетовой окраской отличаются высокой антиоксидантной активностью благодаря содержанию антоцианов в оболочке и представляют интерес для производства полезных продуктов питания. Однако у тритикале пока не выведены формы с богатой антоцианами фиолетовой окраской зерна. Цель настоящей работы – получение с использованием маркер-вспомогательной селекции новых форм пшенично-ржаных амфиплоидов с фиолетовой окраской зерна и сравнительная оценка их по содержанию антоцианов и показателям продуктивности. Определение генотипа гибридов тритикале Садко (× Triticosecale Wittmack) с фиолетовозерной линией полбы 27-3/17 (T. dicoccum (Schrank) Schuebl.) проводили с применением молекулярных ДНК-маркеров. У всех гибридов F3 , имеющих фиолетовую окраску зерна, установлено наличие двух комплементарно действующих доминантных генов, Pp3 и Pp-B1, отвечающих за повышенное содержание антоцианов в зерне. В последующих поколениях растения пшенично-ржаных амфиплоидов имели фиолетовую окраску зерна. Общее содержание антоцианов в цельнозерновой муке гибридов варьировало от 36 до 529.3 мкг/г. Высокое содержание определено у образца 2-1-6-6 (529.3 мкг/г). На уровне контроля (полба – 382.6 мкг/г) были образцы 2-1-1-4е, 2-1- 5-10а и 2-1-6-4б. Растения гибридов F5–6 обладали типичным фенотипом гексаплоидных тритикале. По длине колоса и количеству колосков превышали показатели полбы. Число зерен колоса у гибридов было меньше, чем у контроля тритикале Садко, и достигло в среднем 28.0 и 34.4 шт. в 2024 и 2025 гг. соответственно. Масса 1000 зерен фиолетовозерных семей тритикале в 2025 г. была на уровне материнской формы Садко и составила в среднем 47.6 г. Средняя урожайность с единицы площади у гибридов (470 г/м2) в 2025 г. была выше, чем у полбы (306 г/ м2), но ниже, чем у Садко (584 г/м2). Таким образом, получен селекционный материал фиолетовозерных форм тритикале, по ряду признаков схожий с материнской формой тритикале Садко, но отличающийся от отцовской формы полбы – донора фиолетовой окраски зерна

## Introduction

The artificial allopolyploid triticale (× Triticosecale Wittmack) was developed by combining the genomes of wheat (Triticum sp.) and rye (Secale sp.). This species has existed for less than 150 years. The first fertile octoploid (2n = 8x = 56) plants of wheat-rye amphiploid were made in 1888 by the German researcher W. Rimpau (Muntzing, 1974). They had three doubled common wheat genomes (ВВAuAuDD) and one doubled rye genome (RR). Due to their cytogenetic instability, meiotic disturbances are often observed in primary octoploid triticales resulting in the appearance of aneuploid plants with a reduced chromosome number in the progeny (Silkova et al., 2021). Such plants can reach up to 70 % of the population (Stepochkin, Vladimirov, 1978). Divergent forms of hexaploid (6x) triticales often arise in the progenies of aneuploid plants (Kalinka, Achrem, 2018). They are more productive and cytogenetically stable than octoploid ones, which explains the fact that triticale cultivars worldwide are predominantly hexaploid.

Along with high yield potential and good grain quality, triticale exhibits resistance to many diseases and adverse environmental conditions, making this crop promising for agricultural production. Over recent decades, global triticale production has gradually increased, exceeding 10 million tons (https://www.fao.org/faostat/en/#data/ QCL). The largest producers are Poland, Belarus, Germany, Russia, and France (Hamid et al., 2024).

Triticale is widely used for nutritional and forage purposes, as well as in the production of biofuels, alcohol, and in brewing (Cantale et al., 2016; Mirontseva et al., 2018; Zhu, 2018; Gaviley et al., 2024; Latini et al., 2024). Triticale grain has a higher biological value than wheat and rye. Due to its high lysine concentration, improved protein digestibility, and balanced mineral content, it is used as health-promoting nutrition in the diet of people and animals (Kamanova et al., 2023). Furthermore, triticale is suitable for producing high-quality flour used in bread baking and confectionery industries, including for making dietary bread and breakfast cereals (Leonova et al., 2022).

Despite its many advantages, triticale is still underutilized and consumed in much smaller quantities than other major cereals. This is primarily due to the wrinkled grain, flour color, and specific taste characteristics. Breeders put continuous efforts aiming to improve triticale grain features and successfully create smooth-grained varieties with flour quality not inferior to that of wheat (Camerlengo, Kiszonas, 2023). It is worth noting that the low genetic diversity of triticale confirmed by the studies based on molecular data (Niedziela et al., 2016; Losert et al., 2017) is determined by the gene pool of the parental wheat and rye species. Therefore, it is important to expand the gene diversity of triticale by introducing donors of new traits.

Nowadays, consumers are increasingly striving for healthier diets, making a demand for alternative solutions, when it comes to cereal crops used in human and animal nutrition. Cereals with blue, purple, and black colored grains as well as increased anthocyanin content show enhanced functional and nutritional properties and attract the increasing attention of researchers (Sharma S. et al., 2018; Loskutov, Khlestkina, 2021; Yudina et al., 2021). Anthocyanins are a group of water-soluble polyphenolic compounds that have a positive impact on human health due to their anti-inflammatory, anti-diabetic, anti-cancer, and antibacterial properties (Zhu, 2018; Francavilla, Joye, 2020; Gard et al., 2022).

It is known that purple grain color is determined by the synthesis and accumulation of anthocyanins in the pericarp of the grain. This process is initiated as a result of complementary interaction between two key genes, Pp3 and Рр-1 (Khlestkina, 2013). The Pp3 gene was transferred from T. aethiopicum Jakubz to chromosome 2A of wheat, and the Рр-1 gene is localized on the short arms of homeologous group 7 chromosomes and controls the coloration of the coleoptile and other plant organs (Khlestkina, 2013; Shoeva et al., 2014; Khlestkina et al., 2015).

At present, major efforts are underway in Russia to develop initial breeding material with colored grain in wheat (Khlestkina et al., 2017; Vasilova et al., 2021; Rubets et al., 2022; Shamanin et al., 2022; Gordeeva et al., 2024; Chumanova et al., 2025), barley (Kukoeva et al., 2024), and triticale (Petrash, Stepochkin, 2023). Several purple-grained common wheat cultivars, namely Nadira, Pamyati Konovalova, and Ef 22, have been registered in the State Register of Breeding Achievements (https:// gossortrf.ru).

Creating triticale with increased anthocyanin content in the grain will redefine the development potential of this crop as a product for healthy, balanced, and functional human and animal nutrition. Currently, there are no registered triticale cultivars with purple grain color. Purple-grained forms and lines of wheat and emmer wheat can act as donors of this trait. However, when hybridizing purple-grained soft wheat (genomic formula BBAADD) with hexaploid triticale (genomic formula BBAARR), diploidization of a hybrid may cause the risk of complete or partial loss of R-genome chromosomes in the progeny, leading to the formation of a 6x forms with an incomplete set of rye chromosomes, which is observed in hybrids of triticale with spring common wheat over a number of generations (Badaev et al., 1985).

Crossing purple-grained common wheat with rye can produce haploid sterile hybrids that require colchicine treatment for chromosome doubling to obtain purplegrained octoploid wheat-rye amphiploids. These, in turn, are to be crossed with hexaploid triticale forms to subsequently select 6x amphiploids with purple grain color in the progeny. The example of successful development of new hexaploid-level triticale forms in crosses of 28-chromosome emmer wheat (T. dicoccum (Schrank) Schuebl.) with hexaploid triticale (Silkova et al., 2023) gave an impetus to developing purple-grained triticale as a result of crosses of 6x triticale with the previously obtained emmer wheat line 27-3/17 with a characteristically high anthocyanin content in the grain (Stepochkin et al., 2023).

The goal of the present paper was to obtain new forms of wheat-rye amphiploids with purple grain color using marker-assisted breeding and to comparatively evaluate their anthocyanin content and productivity parameters

## Materials and methods

**Fig. 1.**
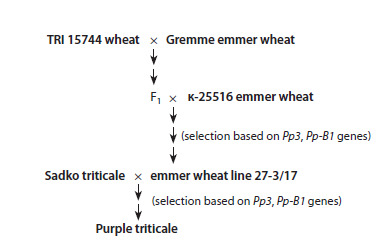
The purple-grain triticale production scheme.

Plant material. F 2 hybrids (Triticosecale × T. dicoccum) were used as plant material. The hybrids created by crossing the hexaploid triticale cultivar Sadko from the global collection of the Vavilov Institute of Plant Genetic Resources (VIR k-3927) and the purple-grained emmer wheat line 27-3/17 (Stepochkin et al., 2023) were grown using the in vitro embryo culture method (Petrash, Stepochkin, 2023). The plant material production scheme is shown in Figure 1.

DNA isolation and molecular analysis. Total DNA was isolated from young leaves of individual plants according to the method described by J. Plaschke et al. (1995). The PCR reaction mixture contained 100 ng of template DNA, reaction buffer (100 mM Tris-HCl, pH 8.5 (at 25 °C), 100 mM KCl, 0.4 mM of each deoxynucleoside triphosphate, 4 mM MgCl2, 0.06 units/μL Taq DNA polymerase, 0.2 % Tween 20, stabilizers for HSTaq DNA polymerase) (“Biolabmix”, Russia), 0.3 μM forward and reverse primers (“Biosset”, Russia). Plant screening was performed using the intragenic marker Pp3-diagnostic (Shoeva et al., 2021) developed for the Pp3 gene on chromosome 2A and the microsatellite marker Xgwm0046 flanking the Pp-B1 gene located on chromosome 7B. PCR procedures with the markers and separation of PCR products in agarose gel were carried out according to the described protocols (Shoeva et al., 2021; Gordeeva et al., 2023).

Determination of total anthocyanin content. Anthocyanin content was determined with modifications according to the principles and methods specified in the publication (Sharma A. et al., 2023). Mature grains of the studied samples were ground using an EM-3A laboratory mill (Russia). Anthocyanins were extracted from whole grain flour in 1 % HCl in methanol for 12 hours at +4 °C. After centrifugation for 25 minutes at 12,000 rpm and +4 °C, the optical density of the extract was measured using a SmartSpecTM Plus spectrophotometer (Bio-Rad Laboratories, Inc., USA) at wavelengths of 530 and 700 nm. The analysis was performed in triplicate for each sample. Anthocyanin content was calculated in terms of the equivalent amount of the most common grain anthocyanidin, namely cyanidin-3-glucoside, using the formula as follows:

**Formula. 1.**
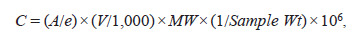
Formula. 1.

where C – the total anthocyanin content, μg cyanidin- 3-glucoside/g flour; A – the calculated absorption value (А530–А700); e – the molar extinction coefficient of cyanidin- 3-glucoside (25.965 cm–1·M–1); V – the total extract volume, mL; MW – the molecular weight of cyanidin- 3-glucoside (449); Sample Wt – the sample weight, mg.

Field evaluation of plant productivity. Study of productivity traits of the developed purple-grained triticale samples and their parents was conducted in 2024 and 2025 on the experimental field of the Siberian Research Institute of Plant Production and Breeding – Branch of the Institute of Cytology and Genetics, Siberian Branch of Russian Academy of Sciences (SibRIPP&B – Branch of IC&G SB RAS). Plants were grown on plots 100 cm wide with 20 cm between rows.

Weather conditions during the years of field experiments were characterized by excessive moisture and high temperatures. By the beginning of September 2024, the sum of effective temperatures was 1,590.8 °C, and in 2025, it was 1,601 °C, compared to the long-term average of 1,428 °C. The moisture supply level of the 2024 growing season was excessive (hydrothermal coefficient (HTC of 1.91 in May–August), with August receiving 2.3 times the normal precipitation, which led to the development of fungal infections and pre-harvest sprouting. The 2025 growing season was generally characterized by sufficient moisture supply (HTC of 1.13 in May–August), with the precipitation being below the long-term average in June and July and above that in August.

Statistical analysis. Statistical analysis was performed using the Statistica 12 and Microsoft Excel 2016 software. Values of plant trait indicators were compared using Student’s t-test.

## Results and discussion

High spike sterility and low germination were noted in early generations of triticale and purple-grained emmer wheat hybrids. Dwarf sterile plants with spikes resembling those of emmer wheat were observed. Completely sterile plants were no longer observed starting from F4. All spikes of plants in the next generation were typical for hexaploid triticale.

**Fig. 2.**
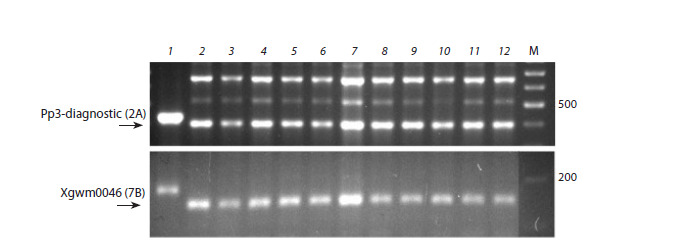
Gel electrophoresis images of PCR products from F4 hybrids as follows: Triticosecale × T. dicoccum and their parental forms with the Pp3-diagnostic and Xgwm0046 DNA markers. 1 – Sadko triticale; 2 – emmer wheat line 27-3/17; 3–12 – F4 hybrids: Sadko × 27-3/17; М – Step 100 DNA marker. Arrows indicate the DNA fragments corresponding to the dominant allele of the Рр3 gene on chromosome 2А (about 398 bp) and the Xgwm0046 marker linked to the dominant allele of the Рр-В1 gene on chromosome 7В (about 186 bp).

Analysis of plants using molecular markers was conducted in F4 hybrids (Fig. 2). All grains in this generation had purple coloration, and the coleoptile had purple color upon germination. According to PCR results with the Pp3-diagnostic marker, it was established that triticale and emmer wheat produced different amplification fragments. As a result of the PCR, all hybrids synthesized a fragment approximately 186 bp in length, which coincided with the fragment from emmer wheat. Therefore, the hybrids carried the dominant Pp3 gene on chromosome 2A.

Analysis with the microsatellite marker Xgwm0046, closely linked to the Pp-B1 gene (responsible for colored coleoptile) showed that the hybrids amplified a fragment of approximately 186 bp in length similarly to the parental emmer wheat, whereas a longer fragment was synthesized with triticale DNA. Based on molecular analysis, it was established that all studied F4 hybrids carried the dominant Pp3 and Pp-B1 genes in a homozygous state.

**Fig. 3.**
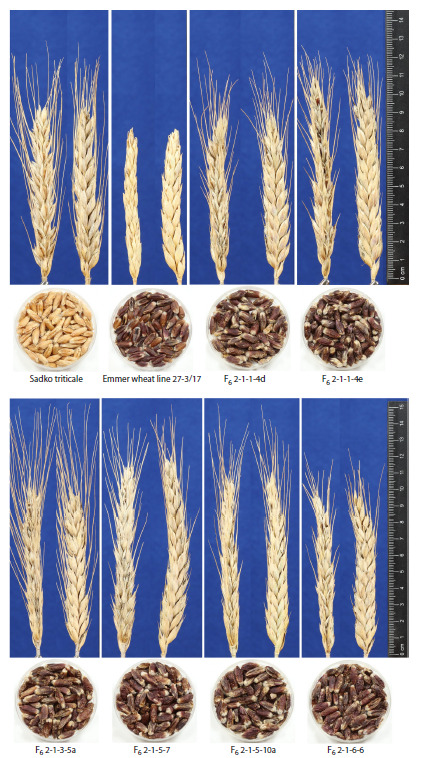
The spikes and grains of purple-grain F6 hybrids Sadko × 27- 3/17 (Triticosecale × T. dicoccum) and their parental forms.

Four best plants (2-1-1; 2-1-3, 2-1-5, and 2-1-6) were selected from the F4 generation based on spike fertility and were subsequently studied as separate families. All plants turned out to be purple-grained in subsequent growing seasons, yet they were morphologically different. In Figure 3, images of the spike (in two projections), as well as grains of some promising F6 hybrids and their parents are presented. It can be seen from the images that the obtained samples had colored grain and an awned spike, morphologically more similar to that of Sadko triticale. It is worth noting that the hybrids, like the maternal cultivar Sadko, had hairy peduncles. This trait is controlled by the dominant Hp gene located on chromosome 5R (Korzun et al., 1996), therefore, the selected triticale forms carried chromosome 5 of rye or its fragment.

The F5–6 progeny was tested for productivity traits under field conditions in 2024–2025 along with the parental forms (see the Table). Structural analysis was performed in 10 typical plants from each family with Sadko triticale used as control. Field testing showed that, similarly to Sadko triticale, the hybrids were resistant to lodging whereas emmer wheat had a weak straw and lodged completely. Unlike emmer wheat, hybrid triticale forms and the Sadko cultivar were also free-threshing cereals with sturdy rachis.

**Table 1.**
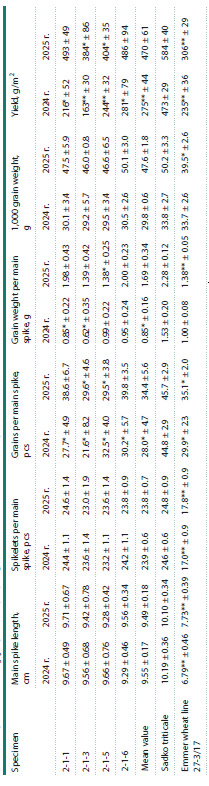
Productivity indicators of F5–6 hybrids of purple-grained triticale forms and their parents in 2024–2025 Note. Mean value is the mean trait value across triticale hybrid forms. Significant differences from Sadko triticale: *p<0.05; ** p<0.01.

In the years of study, the hybrid lines exhibited the spike length and the number of spikelets per spike comparable to those of the Sadko control (over 9 cm and 23 pcs, respectively), whereas emmer wheat spike length did not exceed 8 cm, the number of its spikelets being 17–18 pcs. In terms of the number of grains in the main spike, the hybrids were inferior to the control; significantly lower values were observed for families 2-1-3 and 2-1-5 in 2025 (29.6 and 29.5 pcs, compared to 45.7 pcs in Sadko). Meanwhile, emmer wheat exhibited a significantly lower number of grains than Sadko in both years due to the smaller number of spikelets per spike. The low number of set grains in hybrid triticale forms is associated with partial sterility. The grain weight per spike in hybrid families in the years of study was comparable to the control level but did not exceed it, averaging 0.85 g in 2024 and 1.69 g in 2025; the exception being sample 2-1-3 in 2024 with a significantly lower grain weight per spike at 0.62 g.

The 1,000 grain weight acts as an indicator of grain size; in the years of the study, the obtained purple-grained triticale forms showed 1,000 grain weight values comparable to those of the control cultivar, specifically 29.8 g in 2024 and 47.6 g in 2025 compared respectively to 33.8 g and 50.2 g in Sadko. In terms of yield per square meter, the hybrids were inferior to the control triticale cultivar, especially in 2024, when they showed the average yield of 275 g/m2, compared to 473 g/m2 in Sadko. In 2025, more favorable weather conditions prevailed during the growing season, and the hybrid yield increased to 470 g/m2. The Sadko cultivar also showed a higher result yielding 584 g/m2.

**Fig. 4.**
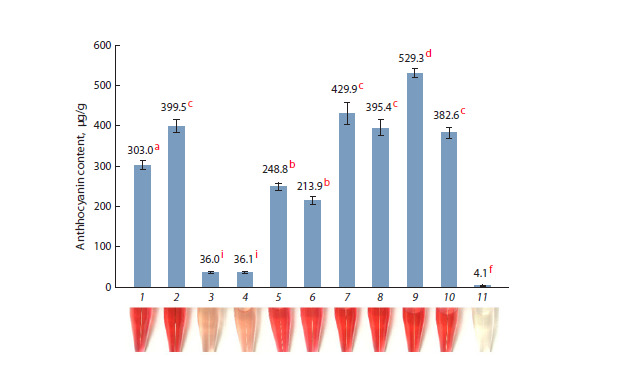
Total anthocyanin content in terms of the equivalent amount of Cy-3-Glu in methanol extracts of whole-grain flour from nine hybrids of purple-grain triticale and their parental forms. 1 – F6 2-1-1-4d; 2 – F6 2-1-1-4e; 3 – F6 2-1-3-3a; 4 – F6 2-1-3-3b; 5 – F6 2-1-3-5a; 6 – F6 2-1-5-7; 7 – F6 2-1-5-10a; 8 – F6 2-1-6-4b; 9 – F6 2-1-6-6; 10 – emmer wheat line 27-3/17; 11 – Sadko triticale. Letters a–f indicate statistically significant differences between the compared groups at p <0.05.

Based on the evaluation of productivity traits, it can be concluded that the developed purple-grained triticale forms exhibited trait values similar to those of Sadko hexaploid triticale. However, most hybrids still showed partial spike sterility possibly related to meiotic disturbances. As shown by previous cytological studies in F6 hybrids of emmer wheat with triticale, meiotic anomalies persisted over several generations (Silkova et al., 2023). It is also known that, when it comes to triticale hybrids, genome stabilization and meiotic normalization can occur even in later generations (Kalinka, Achrem, 2018). moderate to intense. Total anthocyanin content (TAC) was determined in whole grain flour obtained from the 2025 field material. The results of the tested plants were compared to those of emmer wheat line 27-3/17, i. e. the donor of purple coloration (Fig. 4). The obtained data showed that TAC values in hybrids ranged from 36 μg/g to 529.3 μg/g. The control (emmer wheat) value was 382.6 μg/g. A significantly higher value of 529.3 μg/g was observed in specimen 2-1-6-6. The specimens 2-1- 1-4e at 399.5 μg/g, 2-1-5-10a at 429.9 μg/g, and 2-1-6- 4b at 395.4 μg/g showed the results comparable to that of emmer wheat. High TAC values (of over 200 μg/g) were also recorded in samples: 2-1-1-4d, 2-1-3-5a, and 2-1-5-7. The lowest TAC values (36 μg/g) were found in the whole grain flour of the samples 3a and 3b from the family 2-1-3. The white-grained maternal triticale cultivar Sadko showed a total anthocyanin content of 4.1 μg/g flour.

According to estimates by various authors, the total anthocyanin content in purple-grained wheat grain ranges from 16 to 477 μg/g (Gordeeva et al., 2024). In this paper, triticale forms with high anthocyanin content of 529 μg/g were obtained exceeding the results of the donor purple-grained emmer wheat line. It is known that anthocyanin biosynthesis in the grain pericarp requires a complementary interaction of two genes, Pp-1 and Pp3. The latter is a unique gene inherited from an Ethiopian wheat (Khlestkina, 2014). At the same time, the Pp-1 gene controlling the coloration of the coleoptile and other plant organs, is widespread among rare wheat species (Shoeva et al., 2024) and common wheat cultivars (Gordeeva et al., 2024).

Pigmentation of plant organs is widespread among triticale cultivars; for example, the spring triticale cultivar Amore has strong anthocyanin coleoptile coloration (Skatova et al., 2018), cultivars Venets, Ullubiy, and Boguslav exhibit moderate coloration (https://gossortrf. ru). The cultivar description for Sadko triticale discussed in this paper cites the anthocyanin coloration of the flag leaf auricles (Grib et al., 2011). It is possible that Sadko carries one of the Pp-1 gene alleles, which, in combination with the Pp3 and Pp-B1 genes from emmer wheat, determines greater expression of the grain coloration trait in some hybrids.

Obtaining constant forms or lines of wheat-rye amphiploids using hybridization of triticale with emmer wheat is a lengthy process. Its complexity is due to ongoing shaping associated both with the recombination of genes from the two species and with genomic rearrangements. To accelerate the solution of this problem at the next breeding stage, it is important to apply the doubled haploid method based on the obtained purplegrained triticale forms.

## Conclusion

In the present paper, a successful attempt has been made to develop triticale forms with purple grain based on crossing the triticale cultivar Sadko and emmer wheat line 27-3/17 acting as the donor of the Pp3 and Pp-B1 genes responsible for grain color. The obtained hybrid triticale forms have a purple grain color, a high total anthocyanin content, and approach the maternal triticale form in terms of productivity indicators and spike shape. Further research should be aimed at obtaining constant, genetically stable lines of purple-grained triticale, which will serve as initial breeding material and become donors for high anthocyanin content in grain for other triticale cultivars. It is assumed that purple-colored triticale grains will be used as an additional source of antioxidants in functional nutrition for humans and animals.

## Conflict of interest

The authors declare no conflict of interest.
